# Visfatin in the porcine pituitary gland: expression and regulation of secretion during the oestrous cycle and early pregnancy

**DOI:** 10.1038/s41598-023-45255-4

**Published:** 2023-10-25

**Authors:** Karolina Szymanska, Ewa Zaobidna, Edyta Rytelewska, Ewa Mlyczynska, Patrycja Kurowska, Kamil Dobrzyn, Marta Kiezun, Barbara Kaminska, Nina Smolinska, Agnieszka Rak, Tadeusz Kaminski

**Affiliations:** 1 Department of Animal Anatomy and Physiology, Faculty of Biology and Biotechnology, University of Warmia and Mazury in Olsztyn, Oczapowskiego 1A, 10-719 Olsztyn, Poland; 2 Department of Biochemistry, Faculty of Biology and Biotechnology, University of Warmia and Mazury in Olsztyn, Oczapowskiego 1A, 10-719 Olsztyn, Poland; 3 Laboratory of Physiology and Toxicology of Reproduction, Institute of Zoology and Biomedical Research, Jagiellonian University in Krakow, Gronostajowa 9, 30-387 Krakow, Poland; 4 Doctoral School of Exact and Natural Sciences, Jagiellonian University in Krakow, Lojasiewicza 11, 30-348 Krakow, Poland; 5 Department of Zoology, Faculty of Biology and Biotechnology, University of Warmia and Mazury in Olsztyn, Oczapowskiego 5, 10-719 Olsztyn, Poland

**Keywords:** Reproductive biology, Homeostasis

## Abstract

Visfatin is a multifunctional protein which, besides the control of energy homeostasis, seems to be also involved in the regulation of female fertility through the influence on the endocrine hypothalamus-pituitary-gonadal axis, including the pituitary. The aim of this study was to investigate the expression of visfatin mRNA and protein in the anterior (AP) and posterior pituitary lobes of the pig during the oestrous cycle and early pregnancy. In AP, we also examined colocalisation of visfatin with pituitary tropic hormones. Moreover, we aimed to evaluate the in vitro effects of GnRH, FSH, LH, and insulin on visfatin protein concentration and secretion in AP cells during the cycle. The study showed that visfatin is present in all types of porcine pituitary endocrine cells and its expression is reliant on stage of the cycle or pregnancy. GnRH, FSH, LH and insulin stimulated visfatin secretion by AP cells on days 17 to 19 of the cycle, while on days 2 to 3 visfatin release was enhanced only by LH. Summarising, visfatin is locally produced in the pituitary in a way dependent on hormonal milieu typical for reproductive status of pigs. Further research is required to clarify the role of visfatin in the pituitary gland.

## Introduction

The adipose tissue, considered as a place of energy storage, is also an endocrine organ. It secretes a group of hormones called adipokines^1^. One of them, nicotinamide phosphoribosyltransferase (NAMPT), also termed visfatin, was identified in 2005 by Fukuhara et al.^2^. Visfatin is a 56 kDa multifunctional protein which occurs in two forms: extracellular (eNAMPT), acting as a hormone, and intracellular (iNAMPT), which is involved in the synthesis of nicotinamide dinucleotide adenine^3^. No visfatin receptor has yet been identified, but it was suggested that visfatin may bind and activate the insulin receptor (for review see Grolla et al.^4^). Additionally, chemokine receptor CCR5^5^ and toll-like receptor 4^6^ were indicated as potential visfatin receptors. Visfatin plays an important role in the regulation of energy homeostasis, inflammation, cell differentiation^7^ and angiogenesis^8,9^. Visfatin, taking part in the control of energy homeostasis, seems to be also involved in the regulation of female fertility. A positive correlation was noted between the hormone concentration in the ovarian follicular fluid of women and the number of oocytes retrieved^10^. Similarly in mice, visfatin increased the potential of fertility and developmental competence of oocytes^11^. It is possible that this effect is achieved to some extent by the participation of visfatin in the regulation of ovarian steroidogenesis^12–14^. The involvement of visfatin in the regulation of uterine contractions, implantation and placentation is also suggested^15,16^.

It seems that visfatin, apart from its direct effect on the reproductive system, may additionally influence the endocrine hypothalamus-pituitary-gonadal axis (HPG), including the pituitary, what has not been investigated so far. Visfatin is expressed in all structures of the HPG axis: in the hypothalamus of pigs^17^ and mice^18^, in the pituitary gland of mice^19^ and sheep^20^, as well as in the ovarian follicular cells of humans^12^, buffaloes^14^ and pigs^21^. Its expression is hormonally controlled. In human granulosa cells, visfatin gene (*NAMPT*) expression was increased in response to hCG and prostaglandin E_2_^10^. Also in our recent studies on pig luteal cells, we found the effect of luteinising hormone (LH), progesterone (P_4_), insulin (INS), and prostaglandins E_2_ and F_2α_ on visfatin protein expression. The visfatin expression in response to the treatments was dependent on the endocrine milieu related to the oestrous cycle^22^. It seems that also hormonal status related to pregnancy may affect visfatin production, which is strongly suggested by the increase in visfatin plasma concentration with advancing gestational age of woman^23^.

We hypothesised that the expression of visfatin in the pituitary gland is dependent on the hormonal status of animals. Therefore, the aim of this study was to investigate the visfatin gene expression and protein concentration in the anterior (AP) and posterior pituitary (NP) lobes of the pig during the oestrous cycle (days 2 to 3–the early-luteal phase, 10 to 12–the mid-luteal phase, the phase in which the steroidogenic activity of the corpus luteum is the highest throughout the cycle and similar to its activity observed during pregnancy, 14 to 16–the late-luteal phase and 17 to 19–the follicular phase) and early pregnancy (days 10 to 11–the migration of the embryos within the uterus, 12 to 13–the maternal recognition of pregnancy, 15 to 16–the beginning of implantation and 27 to 28–the end of implantation). In AP lobe, we also examined colocalisation of visfatin with LH, follicle-stimulating hormone (FSH), adrenocorticotrophic hormone (ACTH), thyroid-stimulating hormone (TSH), prolactin (PRL) and growth hormone (GH) on days 10 to 12 of the oestrous cycle. Moreover, we aimed to evaluate the in vitro effects of gonadotrophin-releasing hormone (GnRH) – the main regulator of pituitary gonadotrophs, FSH and LH – hormones produced by these cells, and INS – energy homeostatic signal, on visfatin protein concentration and secretion in AP cells (APc) during the oestrous cycle.

## Results

### Gene and protein expression of visfatin in the anterior pituitary gland

**Figure 1:**
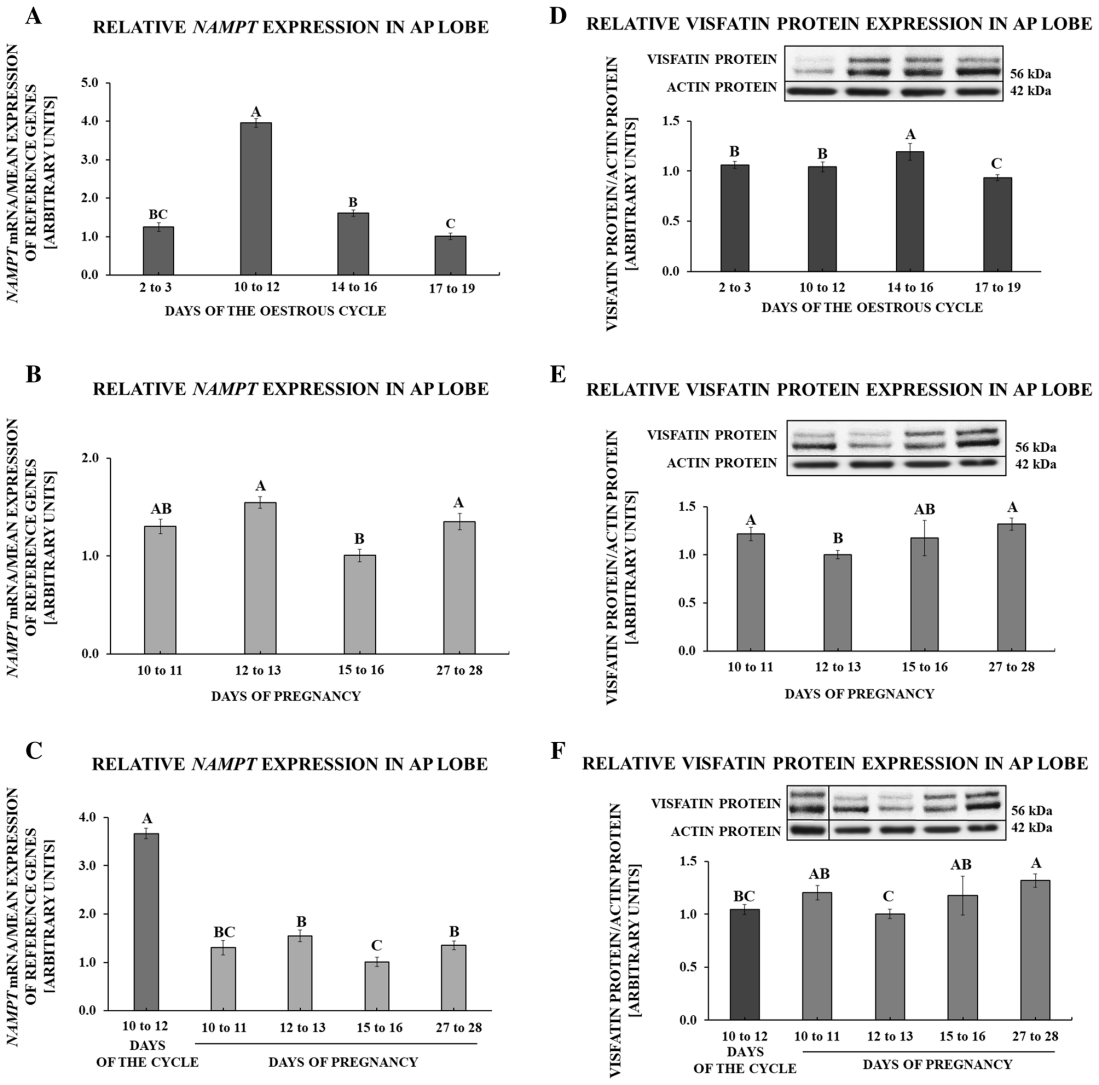
Visfatin gene *(NAMPT)* and protein expression in the anterior pituitary lobe (AP) of pigs. Relative expression of visfatin mRNA and protein determined using quantitative real-time PCR and Western blot procedures, respectively, in AP tissues collected on days 2 to 3, 10 to 12, 14 to 16, 17 to 19 of the oestrous cycle (**A**,**D**), days 10 to 11, 12 to 13, 15 to 16, 27 to 28 of pregnancy (**B**,**E**) and days of early pregnancy compared to days 10 to 12 of the oestrous cycle (**C**,**F**). Right side of the figure: upper panels contain representative immunoblots (all bands shown in the figure come from the same blot; for an additional comparison at point F, where the concentrations of proteins during chosen periods of the cycle/pregnancy were compared, the artificially arranged bands were separated by a vertical line; uncropped images of visfatin and actin immunoblots are attached in the Supplementary File 1), lower panels demonstrate densitometric analysis of expression of visfatin protein relative to actin protein; results are means ± S.E.M (n = 5). Values associated with bars with different superscripts are different (one way ANOVA followed by Tukey post hoc test), capital letters indicate *p* < 0.05.

During the oestrous cycle, the greatest expression of visfatin gene was observed on days 10 to 12 (*p* < 0.001). On days 17 to 19, the expression was decreased relative to days 10 to 12 and 14 to 16 of the cycle (*p* < 0.001; Fig. 1A). During pregnancy, *NAMPT* expression was suppressed on days 15 to 16 in comparison to days 12 to 13 and 27 to 28 (*p* = 0.005; Fig. 1B). Comparing visfatin gene expression throughout the early pregnancy with days 10 to 12 of the oestrous cycle, visfatin mRNA content during all periods of pregnancy was significantly lesser (*p* < 0.001; Fig. 1C).

During the oestrous cycle, the greatest expression of visfatin protein was observed on days 14 to 16, whereas the lowest was on days 17 to 19 (*p* < 0.001; Fig. 1D). During pregnancy, visfatin protein expression was decreased on days 12 to 13 in comparison to days 10 to 11 and 27 to 28 (*p* = 0.002; Fig. 1E). Comparing visfatin protein abundance throughout the early pregnancy with days 10 to 12 of the oestrous cycle, it was found the increase of the protein concentration on days 27 to 28 of pregnancy (*p* < 0.001; Fig. 1F).

### Gene and protein expression of visfatin in the posterior pituitary gland

**Figure 2:**
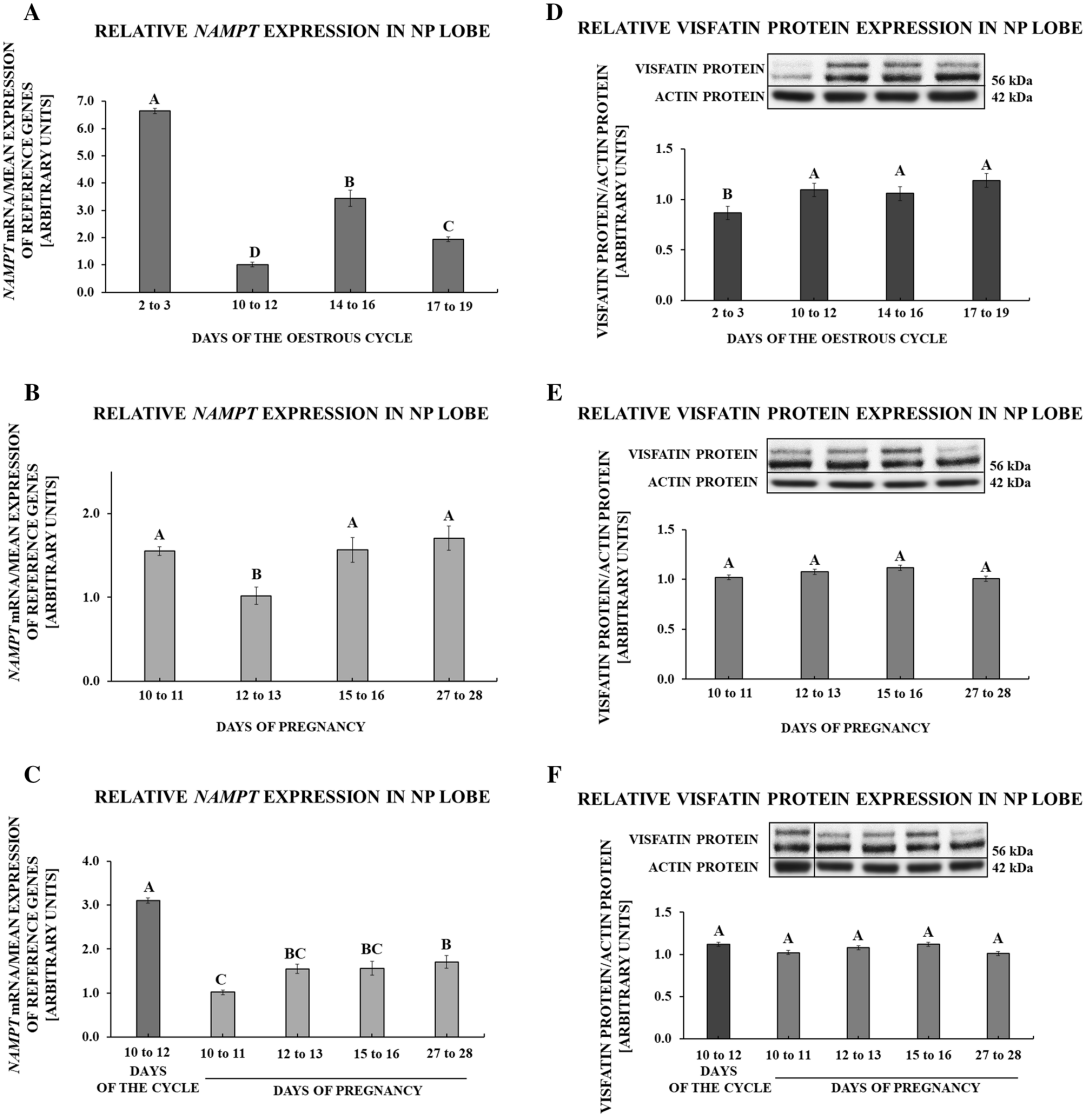
Visfatin gene *(NAMPT)* and protein expression in the posterior pituitary lobe (NP) of pigs. Relative expression of visfatin mRNA and protein determined using quantitative real-time PCR and Western blot procedures, respectively, in NP tissues collected on days 2 to 3, 10 to 12, 14 to 16, 17 to 19 of the oestrous cycle (**A**,**D**), days 10 to 11, 12 to 13, 15 to 16, 27 to 28 of pregnancy (**B**,**E**) and days of early pregnancy compared to days 10 to 12 of the oestrous cycle (**C**,**F**). Right side of the figure: upper panels contain representative immunoblots (all bands shown in the figure come from the same blot; for an additional comparison at point F, where the concentrations of proteins during chosen periods of the cycle/pregnancy were compared, the artificially arranged bands were separated by a vertical line; uncropped images of visfatin and actin immunoblots are attached in the Supplementary File 1), lower panels demonstrate densitometric analysis of expression of visfatin protein relative to actin protein; results are means ± S.E.M (n = 5). Values associated with bars with different superscripts are different (one way ANOVA followed by Tukey post hoc test), capital letters indicate *p* < 0.05.

During the oestrous cycle, the greatest expression of visfatin gene was observed on days 2 to 3 (*p* < 0.001). We observed lesser values on days 14 to 16 and 17 to 19, while the lowest expression of visfatin gene was on days 10 to 12 of the cycle (*p* < 0.001; Fig. 2A). During pregnancy, *NAMPT* expression was decreased on days 12 to 13 relative to other studied stages (*p* = 0.005; Fig. 2B). Comparing *NAMPT* expression throughout the early pregnancy with days 10 to 12 of the oestrous cycle, visfatin mRNA content during all periods of pregnancy was significantly suppressed (*p* < 0.001; Fig. 2C).

During the oestrous cycle, the lowest expression of visfatin protein was observed on days 2 to 3 in comparison to the other days (*p* < 0.001; Fig. 2D). During pregnancy, visfatin protein expression was constant (*p* = 0.076; Fig. 2E). We observed no differences in the protein content of visfatin between days 10 to 12 of the cycle and stages of early pregnancy (*p* = 0.159; Fig. 2F).

### The distribution of visfatin in the porcine pituitary cells

**Figure 3:**
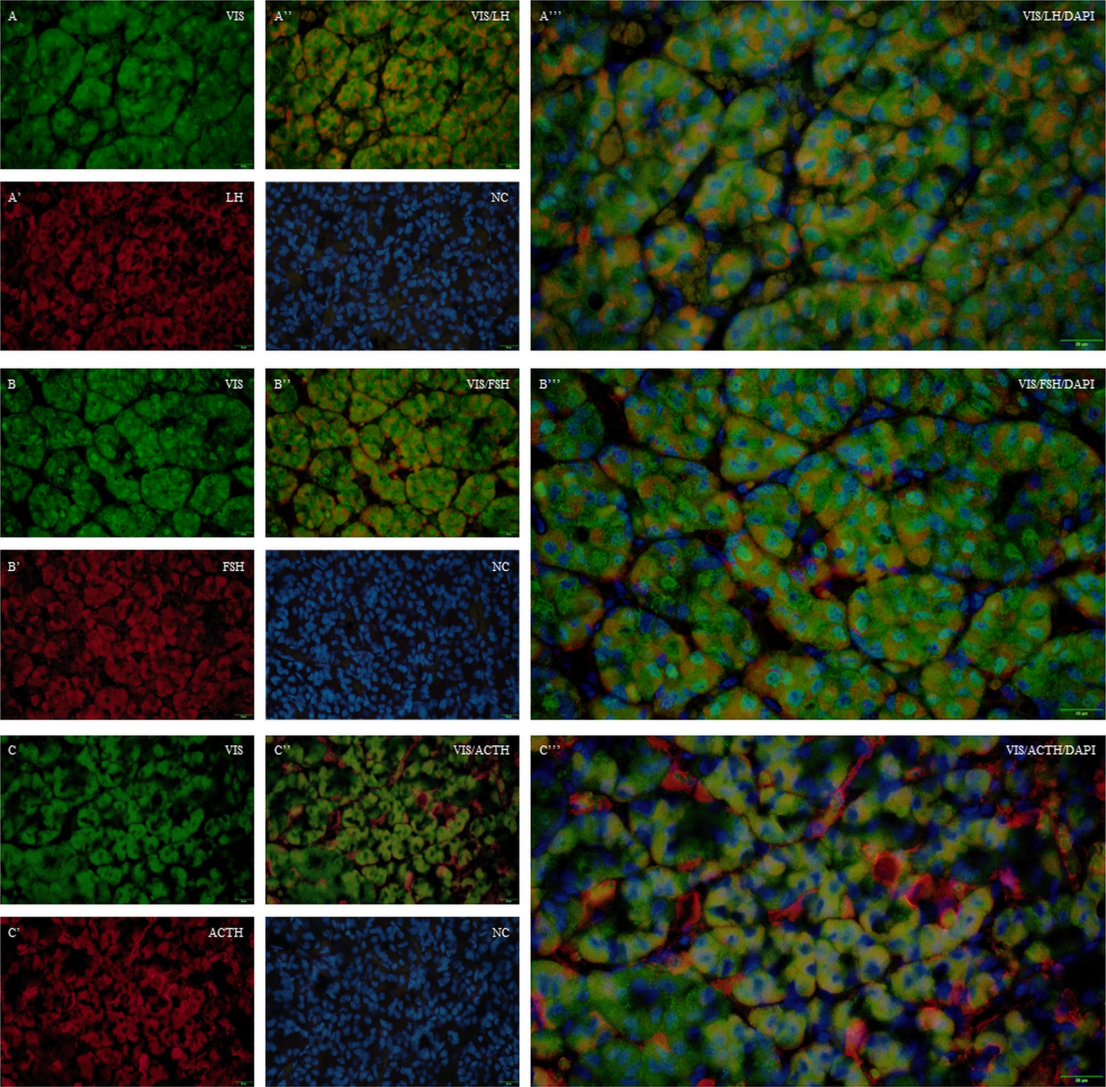
Immunofluorescence localisation of VIS and LH, FSH or ACTH in the porcine pituitary cells collected on days 10 to 12 of the oestrous cycle. Immunofluorescence labelling of VIS, DAPI (nuclear staining, NC) and LH (**A**–**A**’’’), FSH (**B**–**B**’’’), and ACTH (**C**–**C**’’’) in the porcine pituitary on days 10 to 12 of the oestrous cycle. Magnification of 40x, scale bar 20 µm. *VIS* visfatin, *LH* luteinising hormone, *FSH* follicle-stimulating hormone, *ACTH* adrenocorticotrophic hormone, *NC* negative control.

**Figure 4:**
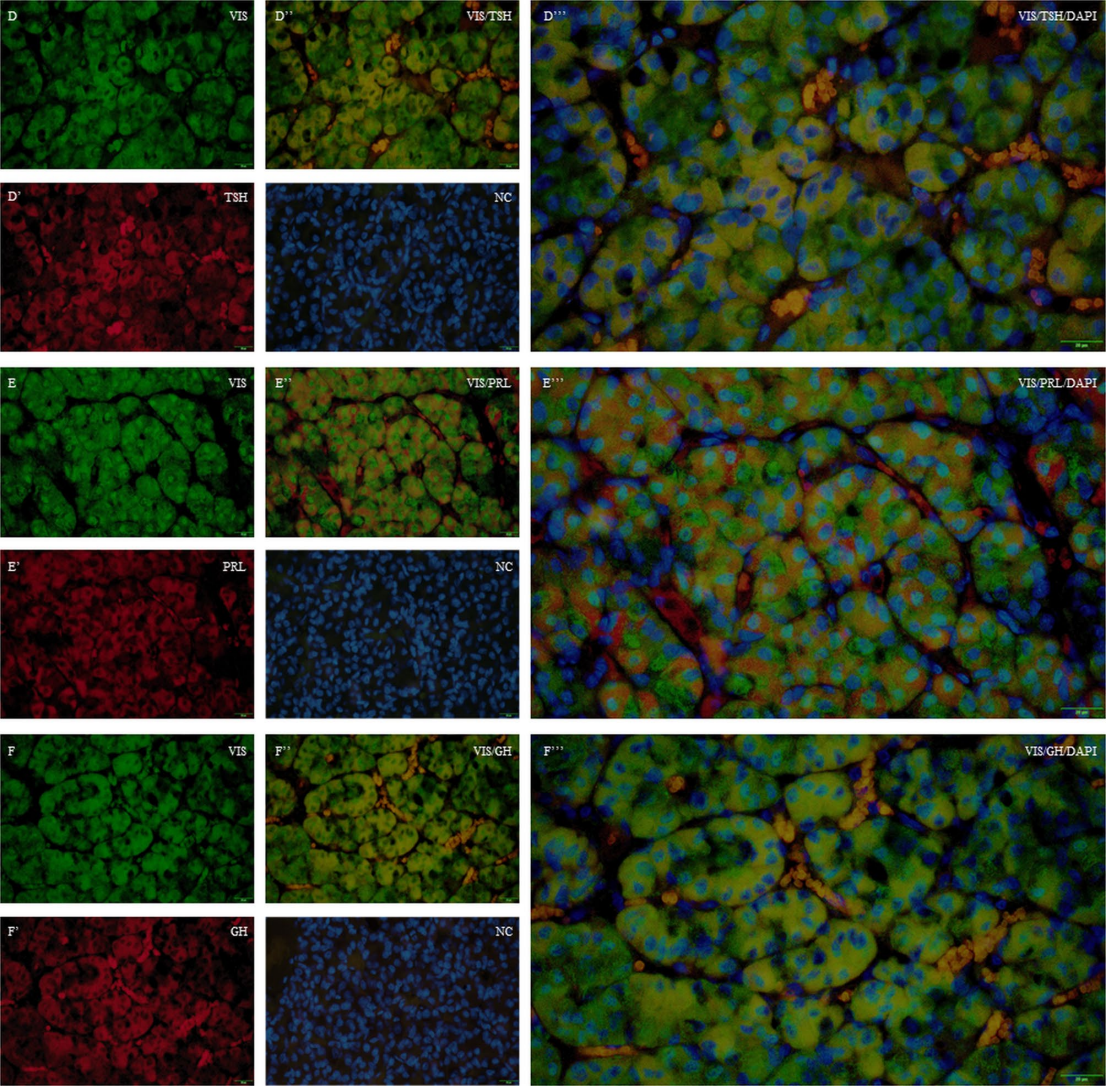
Immunofluorescence localization of VIS and TSH, PRL or GH in the porcine pituitary cells collected on days 10 to 12 of the oestrous cycle. Immunofluorescence labelling of VIS, DAPI (nuclear staining, NC) and TSH (**D**–**D**’’’), PRL (**E**–**E**’’’), and GH (**F**–**F**’’’) in the porcine pituitary on days 10 to 12 of the oestrous cycle. Magnification of 40x, scale bar 20 µm. *VIS* visfatin, *TSH* thyroid-stimulating hormone, *PRL* prolactin, *GH* growth hormone, *NC* negative control.

We confirmed the localisation of visfatin in LH-immunoreactive (IR) cells, FSH-IR cells, ACTH-IR cells, TSH-IR cells, PRL-IR cells, GH-IR cells in the porcine pituitaries collected during the oestrous cycle (days 10 to 12; Figs. 3 and 4).

### Determination of GnRH, FSH, LH and INS impact on visfatin protein expression and secretion by APc during the oestrous cycle

**Figure 5:**
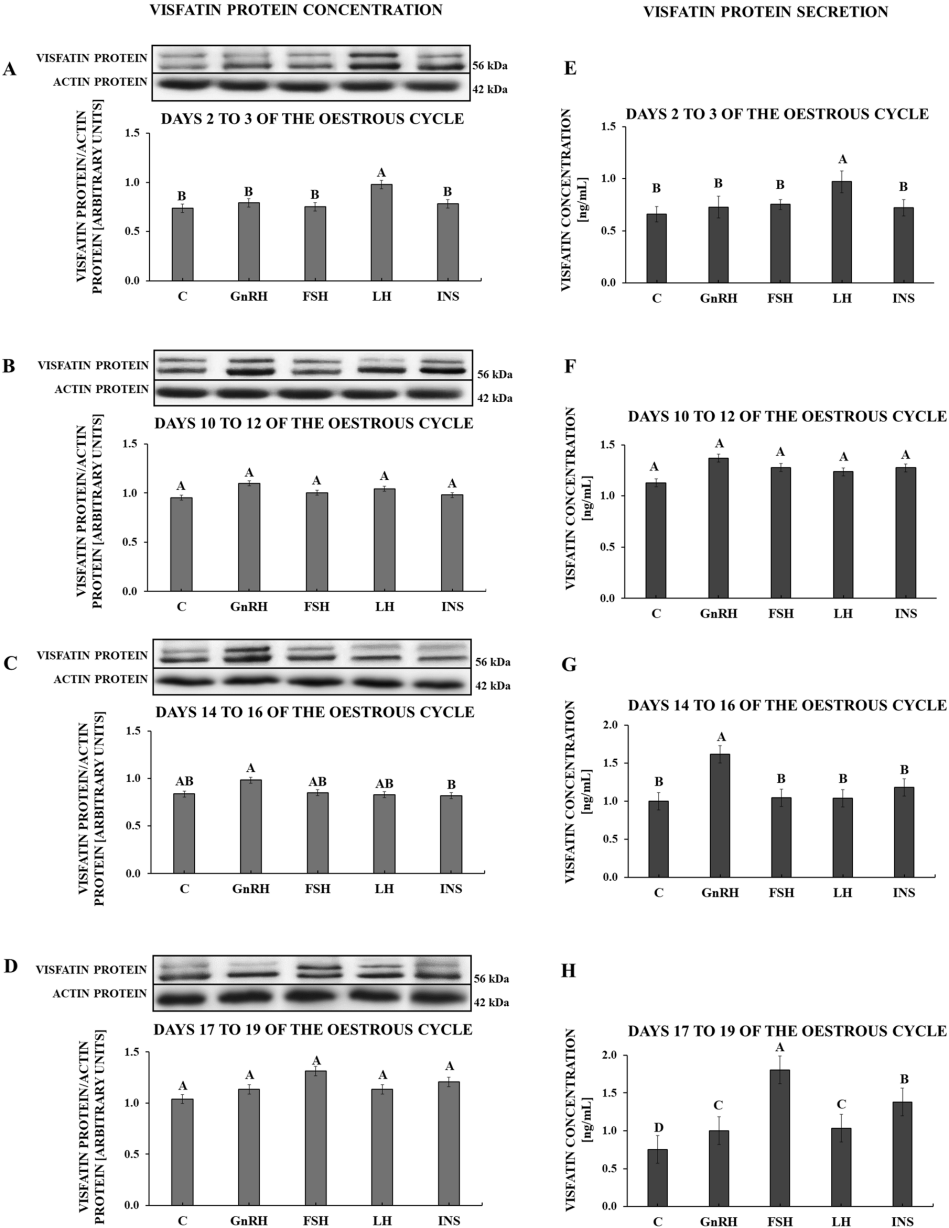
The effect of GnRH (100 ng/mL), FSH (100 ng/mL), LH (100 ng/mL) and INS (10 ng/mL) on visfatin protein expression (**A**–**D**) and visfatin secretion by the porcine pituitary cells (**E**–**H**) during the oestrous cycle. The visfatin protein expression was analysed by Western blot. Results are shown as representative immunoblots (each of the panels represents one blot; uncropped images of visfatin and actin immunoblots are attached in the Supplementary File 1) and bar graphs with densitometry measurement of relative visfatin protein content normalised with actin protein. Visfatin concentration in culture media was evaluated based on ELISA assay. Results are means ± S.E.M (n = 5). Values associated with bars with different superscripts are different; capital letters indicate *p* < 0.05. *C* control, *GnRH* gonadotrophin-releasing hormone, *FSH* follicle-stimulating hormone, *LH* luteinising hormone, *INS* insulin.

On days 2 to 3 of the oestrous cycle, only LH stimulated visfatin protein content in APc (*p* = 0.002; Fig. 5A) as well as visfatin secretion by these cells (*p* < 0.001; Fig. 5E). On days 10 to 12 of the oestrous cycle, none of the tested hormones affected visfatin protein expression (*p* = 0.138; Fig. 5B) and visfatin secretion (*p* = 0.120; Fig. 5F). On days 14 to 16 of the oestrous cycle, none of the tested hormones had any influence on the content of visfatin protein (*p* = 0.031; Fig. 5C), but only GnRH had the stimulatory effect on visfatin secretion (*p* < 0.001; Fig. 5G). On days 17 to 19 of the oestrous cycle, no influence of the tested hormones on the content of visfatin protein in APc was observed (*p* = 0.057; Fig. 5D). However, each of these hormones stimulated visfatin secretion (*p* < 0.001; Fig. 5H).

## Discussion

The presented research was the first experiment to report the expression of visfatin gene and concentration of visfatin protein in the porcine pituitary during the oestrous cycle and early pregnancy, as well as visfatin cellular localisation in this gland. Moreover, we indicated the impact of GnRH, FSH, LH and INS on visfatin protein expression in the in vitro cultured anterior pituitary cells and visfatin secretion by these cells.

We noted that the highest visfatin mRNA abundance was observed in AP lobe during the mid-luteal phase, while in NP, the highest *NAMPT* expression was observed during the early-luteal phase of the cycle. Moreover, our study demonstrated the highest concentration of visfatin protein in AP lobe during the late-luteal phase and the lowest during the follicular phase of the oestrous cycle. This, in turn, implies that visfatin expression is tissue-specific and dependent on the phase of the oestrous cycle. Interestingly, visfatin was not detected in NP of the mouse pituitary and dependence between AP visfatin expression and the phase of the cycle was not found in this species^19^ which suggests that this phenomenon is also species dependent. The porcine pituitary gland, thanks to the presence of both classical oestrogen and progesterone receptors and non-classical membrane receptors, is sensitive to the action of oestradiol (E_2_) and P_4_^24^. Approximately 70% of pituitary gonadotrophs, responsible for LH and FSH generation, express oestrogen receptors in different species^25–27^. Of note, the sensitivity of the gland to steroids changes throughout the cycle. The study by Diekman and Anderson^28^ indicated that the number of cytoplasmic oestrogen and P_4_ receptors in the porcine pituitary is many-fold lower on day 18 compared to days 1 to 15 of the cycle. Thus, the observed in our study variations in transcript and protein levels of visfatin in porcine pituitary lobes could result from the hormonal status of animals related to plasma steroids’ concentrations and the gland sensitivity to them.

The visfatin gene and protein expression patterns in AP and NP are different, in particular during the oestrous cycle, which suggests that the regulation of visfatin synthesis and secretion in both pituitary lobes is also different. AP and NP lobes are morphologically and functionally distinct, but it is known that NP affects AP functioning. While AP lobe is populated mainly by six types of cells: somatotrophs, gonadotrophs, lactotrophs, thyrotrophs, corticotrophs, and folliculostellate cells^29^, the posterior pituitary includes pituicytes that affect AP hormone secretion by releasing cytokines, growth factors and other neuroactive compounds^30^ and it also consists of oxytocin- and vasopressin-secreting neuroendocrine terminals. The ability of porcine NP to synthesize other adipokines, such as adiponectin^31^ and chemerin^32^ is known, but there is no information, apart from the current work, on the possibility of visfatin synthesis. This requires further in-depth research.

During early pregnancy, both visfatin gene and protein expression in AP and NP were relatively constant. It pays attention, however, decreased *NAMPT* expression in the pituitary lobes from pregnant animals compared to days 10 to 12 of the cycle. It is worth noting that according to Diekman and Anderson^28^, the number of oestrogen and P_4_ receptors in the pituitaries of pregnant pigs (days 5 to 15) is essentially lower that during the cycle, indicating lesser sensitivity of the gland to sex steroids. It cannot be ruled out that one of factors controlling the number of pituitary steroid receptors can be also visfatin itself. Such an effect of visfatin on oestrogen receptors α and β (ERα and ERβ) was observed in the mice ovary^33^.

In addition to the mentioned earlier, presumed effect of visfatin on the pituitary expression of steroid receptors, the adipokine is also able to regulate its own synthesis. It was found that in the process of the circadian rhythm control of some cell functions, *NAMPT* gene transcription is regulated by a feedback loop involving visfatin itself^34,35^. The exposure of rat pituitary gland explants to visfatin increased the concentration of *NAMPT* mRNA^36^. Thus, it seems that in pituitary cells, the process of autoregulation of visfatin synthesis is active and it is one of elements controlling the final expression of the adipokine.

Relationship between the visfatin expression and the phase of the oestrous cycle was also noted in other structures of the HPG axis and organs of the reproductive system. Changes in the expression of visfatin in the porcine hypothalamus during the oestrous cycle and early pregnancy were reported in our earlier study^17^. Visfatin gene and protein expression in the hypothalamic structures involved in GnRH synthesis, mediobasal hypothalamus and preoptic area, were dependent on hormonal status related to the phase of the oestrous cycle or early pregnancy and affected by steroid hormones. Analogous observations pertaining to the phase-specific visfatin expression have been made for the ovary of pigs^22^, mice^37^ and water buffaloes^14^. Visfatin protein expression in the porcine corpora lutea was regulated by LH, P_4_, INS, and prostaglandins E_2_ and F_2α_^22^. Similarly, in the mouse uterus, visfatin expression was steroid dependent with stimulatory effect of E_2_ and inhibitory of P_4_^37^.

In this study, we also examined the colocalisation of visfatin protein with LH, FSH, ACTH, TSH, PRL and GH. We observed the presence of visfatin in gonadotrophs, corticotrophs, thyrotrophs, lactotrophs and somatotrophs. Maillard et al.^19^ confirmed visfatin protein localisation in the anterior and intermediate parts of female mice, mainly in gonadotrophic cells, but not in NP lobe. The presence of visfatin in these cells may suggest its auto/paracrine influence on pituitary cells’ functions.

In the next part of the experiment, we examined the effect of GnRH, FSH, LH and INS on the content and secretion of visfatin protein in AP during the oestrous cycle noting the phase-dependent influence of these hormones. The stimulatory effect of GnRH on visfatin secretion, but no content, was observed on days 14 to 16 and 17 to 19 of the cycle, when P_4_ plasma level is decreased and E_2_ enhanced. It is known that P_4_ is a negative regulator of pituitary GnRH receptors^38–41^, whereas E_2_ increased the receptor expression^39,42–44^. Of note, GnRH receptors have been identified in gonadotrophs, thyrotrophs and somatotrophs^45^ and the number of gonadotrophs containing ERα, and, consequently, sensitivity to oestrogen action, increases in the follicular phase of the cycle^46^. It is suggested, however, that the most notable factor inducing GnRH receptors’ expression is GnRH itself (for review see Stamatiades and Kaiser^47^). Thus, the increased number of pituitary GnRH receptors in the follicular phase in different species, including the pig^48^, may result mainly from enhanced plasma GnRH concentration and pulse frequency. In addition, insulin can directly promote gonadotrophs’ response to GnRH stimulation^49–51^. Insulin, apart from its own receptors, is able to bind also insulin-like growth factor 1 (IGF-1) receptors^52,53^, which number increases in the rat pituitary at prooestrous^54^. This may justify to some extent the GnRH action seen at the end of the cycle and not in the early- and mid-luteal phase. The effect of GnRH on visfatin secretion seems to be limited to certain types of APc and takes place in the strictly defined physiological status.

In this study, LH stimulated the content and secretion of visfatin protein in the early-luteal phase. Moreover, FSH and LH enhanced the adipokine secretion in the follicular phase, which may be related to the high physiological blood plasma concentration of both gonadotrophins at the turn of two cycles^55^. The influence of pituitary hormones, including FSH and LH, on visfatin secretion by this gland has been completely unknown until now. It seems however, that there are physiological conditions for such an effect to occur. FSH and LH receptors are localised first of all in gonadal tissues, nevertheless the extragonadal localisation of both receptors in different species was indicated^56–60^. This also applies to the pituitary gland^60–63^. It is suggested the direct LH effect on AP through auto feedback action^64,65^. The ability of LH to down-regulate its own receptors is known^66^. It therefore seems possible that locally produced pituitary hormones, such as FSH and LH, auto/paracrinaly, in a phase-dependent manner, are able to influence visfatin secretion. The presented research should be an impulse for further studies of the impact of gonadotrophins and other pituitary tropic hormones on the gland secretory activity.

It is known that APc, including gonadotrophs, constitute target cells of INS, which may regulate their secretory functions^67^ acting as energy homeostatic signal^50^. Both INS and IGF-1 receptors are present in APc of different species^50,51,68,69^. The deletion of insulin receptor substrate-2, a component of the insulin/IGF-1 signalling cascade, causes female infertility through, i.a., disturbances in pituitary functions, leading to reduced numbers of gonadotrophs and decreased LH concentration^70^. As in the case of GnRH, reproductive status influenced the visfatin response to INS. Its effect on the visfatin secretion was observed in this study during the follicular phase. It seems plausible, that the reason for this is mentioned earlier an increase in IGF-1 receptors’ concentration observed in the phase of plasma E_2_ domination^54^. Oestrogens can enhance IGF-1 receptors’ concentration in APc of different species, including the pig^71^. Moreover, feedback mechanism between IGF-1 and E_2_ is suggested. Oestrogens may sensitise APc to IGF-1 and IGF-1 can up-regulate E_2_ receptors’ expression^52^. The insulin influence on visfatin secretion was also noted in other tissues – human adipocytes increased the adipokine release in response to INS^72^.

It is worth mentioning that the effect of GnRH, gonadotrophins and INS on visfatin protein expression and its secretion was usually different. While the adipokine secretion in the follicular phase was stimulated by these hormones, the protein abundance was unaffected by them. This effect could result from the existence of two forms of visfatin, iNAMPT and eNAMPT^29^, that could be regulated differently. What is more, the extracellular form is approximately 1% of the total NAMPT (for review see Carbone et al.^73^). Since the observed effect of the studied factors is more related to the extracellular form of visfatin, it seems that the physiological response of APc to their action is mainly the release of visfatin acting as a hormone, and not a change in the expression of visfatin protein acting as an enzyme.

The role of visfatin in the pituitary is known to a very limited extent. It was found the inhibitory influence of visfatin on LH secretion by the LβT2 gonadotroph cell line. Moreover, in the same study by Maillard et al.^19^, the increase of LβT2 cells’ proliferation, in response to visfatin, was noted. Other studies using rat corticotrophs have shown that visfatin is able to stimulate ACTH release both directly and indirectly, by the enhancement of interleukin-6 release from folliculostellate cells of the pituitary gland^74^. These results may explain the observed earlier by the same research team increase of pituitary proopiomelanocortin (ACTH precursor) mRNA concentration under the influence of visfatin^36^. Generally however, the role of visfatin in the pituitary, especially its extracellular form, is almost completely unexplored and requires further studies.

The study has shown the dependence of visfatin protein expression in the pituitary on the phase of the oestrous cycle or stage of pregnancy. The visfatin secretion by APc was affected by GnRH, FSH, LH and INS. The obtained results suggest that visfatin is locally produced in the porcine pituitary in a way reliant on hormonal milieu typical for the reproductive status of pigs. Further research is required to clarify the role of visfatin in the pituitary gland.

## Methods

### Experimental animals and tissue collection

Pigs bound for commercial slaughter and meat processing were used in this experiment. Experimental animals were mature cross-breed gilts at the age of 7–8 months and the body weight of 130–150 kg. The diet of the animals was in line with the Polish nutritional standards for domestic pigs. Gilts were monitored daily for oestrus behaviour in the presence of a boar. The phase of the oestrous cycle was also confirmed based on the ovarian morphology characteristic according to Akins and Morrissette^75^.The day of the onset of the second oestrus was marked as day 0 of the oestrous cycle. Natural insemination was performed on days 1 to 2 of the cycle. The stage of pregnancy (days 15 to 16 and 27 to 28) was additionally confirmed based on the morphology of conceptuses/trophoblasts^76^. A few minutes after the slaughter, the pituitary glands (separated into AP and NP lobes) were frozen in liquid nitrogen and stored at 80 °C until processing for RNA and protein isolation. The samples assigned to both the quantitative real-time PCR (qPCR) and Western blot were collected from the same animals at the same time. AP glands utilised for in vitro cell culture were collected and placed in the ice-cold Dulbecco’s Phosphate-Buffered Saline with 100 IU/mL penicillin and 100 μg/mL streptomycin, and transported to the laboratory on ice. Additionally, the pituitaries obtained on days 10 to 12 of the cycle, intended for immunofluorescent staining, were placed in 4% buffered paraformaldehyde (pH = 7.4, 4°C).

### Isolation of APc and in vitro cultures

APc were isolated according to the method described by Kiezun et al.^31^ with modifications. In brief, isolation of the cells was performed through the digestion of the pituitary lobes with 0.2% collagenase (Merck, USA) at 37 °C for 30 min, and then the cells were digested with 0.2% collagenase and 0.25% pancreatin (Merck, USA) in cycles of 10 min until the whole tissue was dispersed. The remaining steps of the procedure and cell preincubation were carried out in accordance with the indicated reference. After preincubation, the media were removed and the cells were rinsed with fresh serum-free McCoy’s 5A medium. The cells were incubated for another 24 h (37 °C, 5% CO_2_ and 95% air) in the presence of the treatments: GnRH (100 ng/mL), FSH (100 ng/mL), LH (100 ng/mL), or INS (10 ng/mL). The cells cultured with medium alone were used as control samples. Insulin and GnRH concentrations were determined based on Kiezun et al.^31^ and Gavin et al.^77^, respectively. Concentrations of FSH and LH were chosen based on Gregoraszczuk et al.^78^. The potential effects of treatments on the cells’ viability were determined using the Alamar Blue test, which revealed that the cultured APc were not affected by the applied treatments. After incubation, media were collected and centrifuged at 800×*g*, supernatants were collected and stored at -20 °C, and the cells were used to isolate the total protein.

### Total RNA isolation, cDNA synthesis and quantitative real-time PCR

RNA isolation, cDNA synthesis and qPCR reactions were performed as described previously^17^. In the case of qPCR, the conditions and characteristics of primers used in the study are detailed in Table 1. Constitutive expression of reference genes (*UBC* and *18sRNA)* was confirmed statistically. Relative gene expression of visfatin was calculated using the 2^−ΔΔCT^ method according to Livak and Schmittgen^79^.

**Table 1:** Primers specification, reaction mixture composition and reaction conditions used in the study for the quantitative real-time PCR.

Gene symbol	Primer sequence	Accession number	Reaction mixture composition	Reaction conditions	Reference
*NAMPT*	F: 5′-CCAGTTGCTGATCCCAACAAA-3′ R: 5′-AAATTCCCTCCTGGTGTCCTATG-3′	XM_003132281.5	Power SYBR Green—12.5 µL cDNA—20 ng Forward primer—300 nM Reverse primer—300 nM H_2_O—up to a total volume of 20 μL	Activation and initial denaturation: 95 °C, 10 min 40 cycles of: denaturation: 95 °C, 15 s annealing: 60 °C, 1 min	^80^
*UBC*	F: 5′-GGAGGAATCTACTGGGGCGG-3′ R: 5′-CAGAAGAAACGCAGGCAAACT-3′	XM_003483411.3	Power SYBR Green—12.5 µL cDNA—20 ng Forward primer—400 nM Reverse primer—400 nM H_2_O—up to a total volume of 20 μL	Activation and initial denaturation: 95 °C, 10 min 40 cycles of: denaturation: 95 °C, 15 s annealing: 60 °C, 1 min elongation: 72 °C, 1 min	^81^
*18sRNA*	F: 5′-TCCAATGGATCCTCGCGGAA-3′ R: 5′-GGCTACCACATCCAAGGAAG-3′	AY265350.1			

*NAMPT* visfatin, *UBC* ubiquitin C, *18sRNA* 18 s ribosomal RNA, F forward primer, R reverse primer.

### Western blot

Western blot analysis was conducted similarly as described previously^17^. Protein isolation was performed using Tissue Protein Extraction Reagent (T-PER; Thermo Fisher Scientific, USA) according to the manufacturer's instructions. Then, samples in the amount of 30 µg of protein (per sample) were used for SDS-PAGE electrophoresis. Protein transfer was performed by semi-dry technique, and blots were blocked with 5% BSA. Specification of antibodies used in the study are detailed in Table 2. The actin protein was used as the reference protein. Constitutive accumulation of actin protein was confirmed statistically. The immunocomplexes were visualised using Immobilon Western Chemiluminescent HRP Western blotting Luminol Reagent (Advansta Inc., USA) and archived using the Chemidoc™ XRS + System (BioRad Laboratories Inc., USA). Specific bands were quantified using the densitometer and ImageJ software (US National Institutes of Health, USA). Data were expressed as the ratio of visfatin protein relative to actin proteins in arbitrary optical density units.

**Table 2:** Specifications of antibodies used in the study for the Western blot analysis.

	Antibodies	Host species	Supplier and catalog number	Dilution
Primary	Anti-visfatin	Rabbit	Abcam, UK; cat. no. ab233294	1:700
	Anti-β-actin	Mouse	Merck, USA; cat. no. A5316	1:5000

	Antibodies	Type of conjugated enzyme	Supplier and catalog number	Dilution
Secondary	Goat anti-rabbit	HRP	Cell Signaling Technology, USA; cat. no. 7074	1:1000
	Horse anti-mouse	HRP	Cell Signaling Technology, USA; cat. no. 7076	1:1000

*HRP* horseradish peroxidase.

### The analysis of visfatin localisation in the porcine pituitary gland using fluorescent immunohistochemistry

Fluorescent immunohistochemistry analysis of colocalisation was conducted on 5 μm thick paraffin pituitary sections (n = 3). To expose the antigen epitopes, sections were boiled with Antigen Retrieval Solution. For the reduction of nonspecific background staining, the sections were incubated with 50 mM NH_4_CL in phosphate-buffered saline (PBS). Subsequently, sections were permeabilised using 0.1% Triton™ X-100 and the nonspecific sites of antibodies binding were blocked through 1.5 h incubation with Fish Serum Blocking Buffer (cat. no. 37527; Thermo Fisher Scientific, USA). Next, the slides were rinsed with 0.1 M PBS and incubated with proper antibodies (specification of antibodies used in the study are detailed in Table 3). For negative control, the primary antibodies were omitted, and slides were incubated with 0.1 M PBS. The nonspecific background noise was reduced through 20 min. incubation of slides with 0.5% Sudan Black B dissolved in 70% EtOH. The obtained sections were air-dried and covered with histology mounting medium Fluoroshield™ (cat. no. F6057; Merck, USA) with DAPI for nuclear counterstaining. The labelled sections were analysed with the use of Olympus BX51 research microscope (Olympus, Japan) equipped with an EXFO x-CiteSeries 120Q fluorescence illuminator (Excelitas Technologies Corp., USA) using appropriate filters set for DAPI and Alexa Fluor® dyes. Images were acquired with the use of Nikon DS-Qi2 microscope digital camera (Nikon, Japan) and NIS-Elements (v. 5.10) imaging software (Nikon, Japan).

**Table 3:** Specifications of antibodies used in the study for the fluorescent immunohistochemistry.

	Antibodies	Host species	Supplier and catalog number	Dilution
Primary	Anti-visfatin	Rabbit	Abcam, UK; cat. no. ab233294	1:150
	Anti-LH	Mouse	Abcam, UK; cat. no. ab212578	1:200
	Anti-FSH	Mouse	Abcam, UK; cat. no. ab233866	1:150
	Anti-ACTH	Mouse	Abcam, UK; cat. no. ab212736	1:500
	Anti-TSH	Mouse	R&D, USA; cat. no. MAB57941	1:200
	Anti-PRL	Mouse	Abcam, UK; cat. no. ab11301	1:200
	Anti-GH	Mouse	Abcam, UK; cat. no. ab218405	1:200

	Antibodies	Type of conjugated fluorophore	Supplier and catalog number	Dilution
Secondary	Goat anti-rabbit	Alexa Fluor® 488	Jackson ImmunoResearch Labs, UK; cat. no. 111-545-003	1:1000
	Goat anti-mouse	Alexa Fluor® 594	Jackson ImmunoResearch Labs, UK; cat. no. 115-585-003	1:1000

*LH* luteinising hormone, *FSH* follicle-stimulating hormone, *ACTH* adrenocorticotrophic hormone, *TSH* thyroid-stimulating hormone, *PRL* prolactin, *GH* growth hormone.

### ELISA assay

The concentration of visfatin in the culture medium was determined using a commercial ELISA kit (cat. no. MBS736963; MyBioSource, USA) as described previously^17^. The intra- and inter-assay coefficients of variability for performed analysis were 6.03% and 6.17%, respectively.

### Statistical analysis

Statistical analyses were performed using Statistica program (StatSoft Inc., USA). All data were tested for the assumption’s normality (Shapiro–Wilk test) and homogeneity of variances (Levene's test), and analysed by one-way ANOVA followed by the Tukey’s honest significance post hoc test. Data were presented as means ± S.E.M. from five independent observations. Values for *p* < 0.05 were considered statistically significant.

### Ethics declarations

The studies were carried out following the Polish Act on the Protection of Animals Used for Scientific or Educational Purposes of the 15th of January 2015 (Journal of Laws Dz. U. 2015 No. item 266) as well as Directive 2010/63/EU of the European Parliament and the Council of the 22nd of September 2010 on the protection of animals used for scientific purposes. Hence, this study did not require the consent of the competent ethics committee for animal experiments.

### Supplementary Information

Supplementary Information.

## Data Availability

The data underlying this article will be shared on reasonable request to the corresponding author.
