# Supplementary material for: Visfatin in the porcine pituitary gland: expression and regulation of secretion during the oestrous cycle and early pregnancy
Source: Sci Rep. 2023 Oct 25;13:18253. doi: 10.1038/s41598-023-45255-4 (PMC10600231; doi:10.1038/s41598-023-45255-4)

VISFATIN PROTEIN ABUNDANCE IN THE ANTERIOR PITUITARY

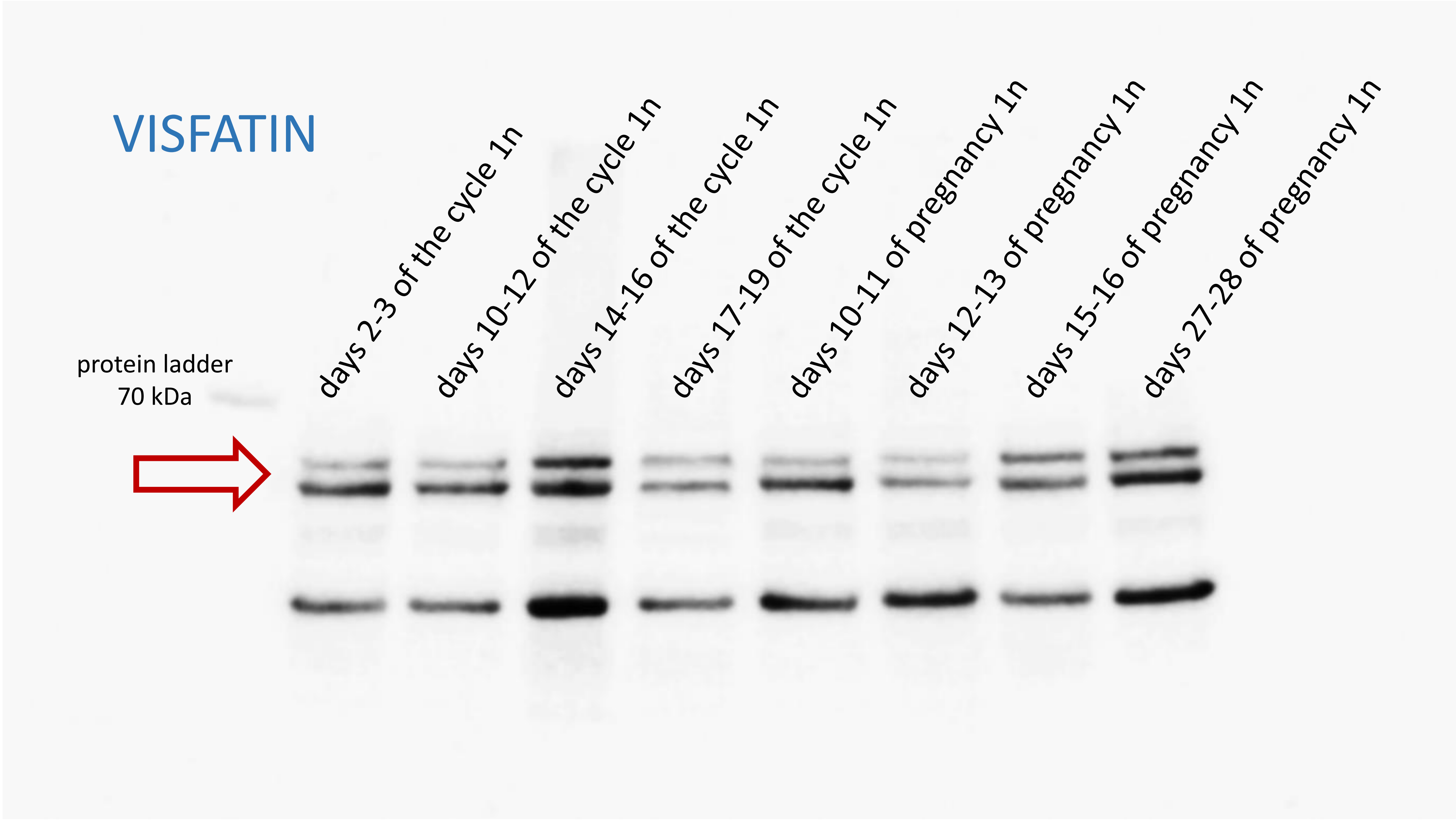

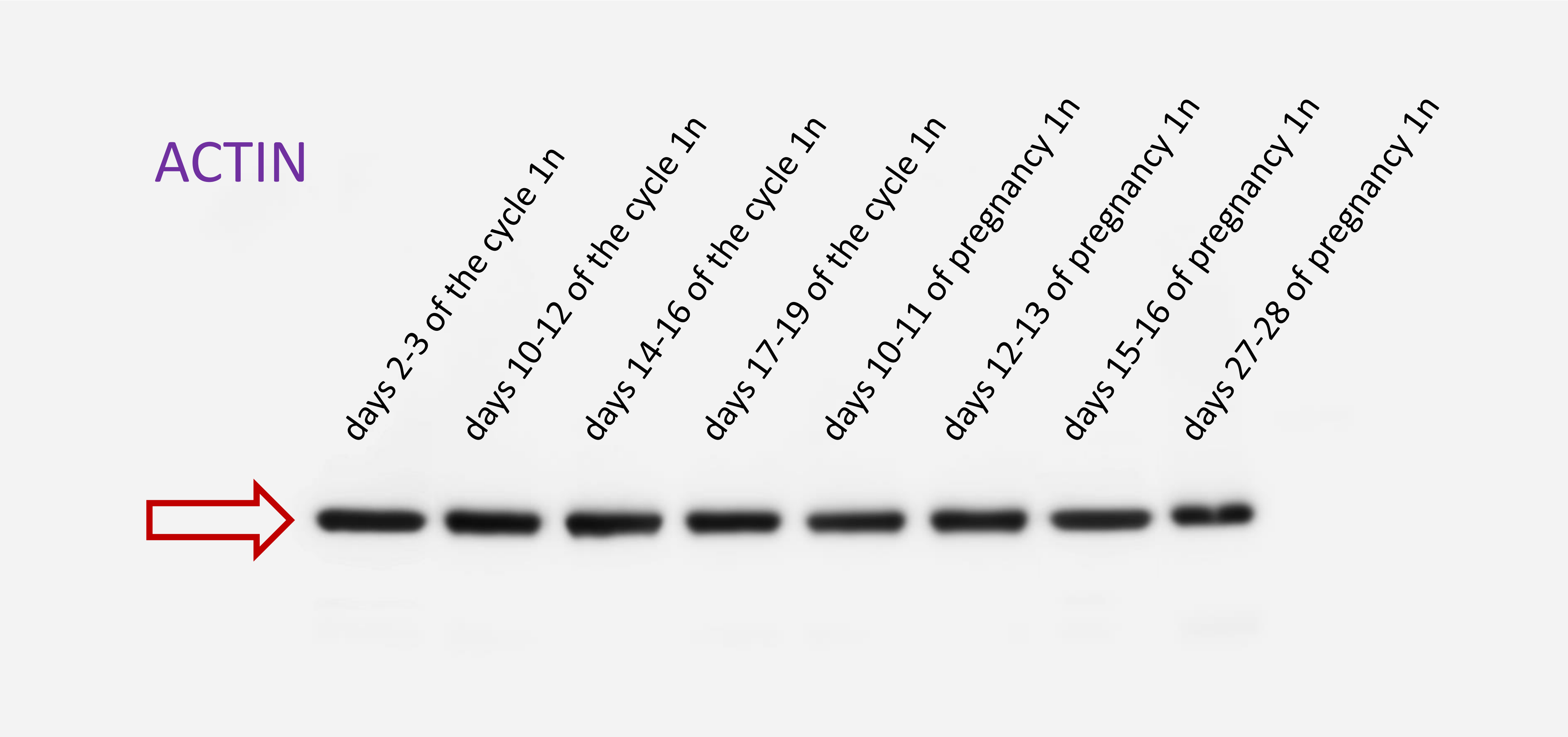

VISFATIN PROTEIN ABUNDANCE
IN THE POSTERIOR PITUITARY

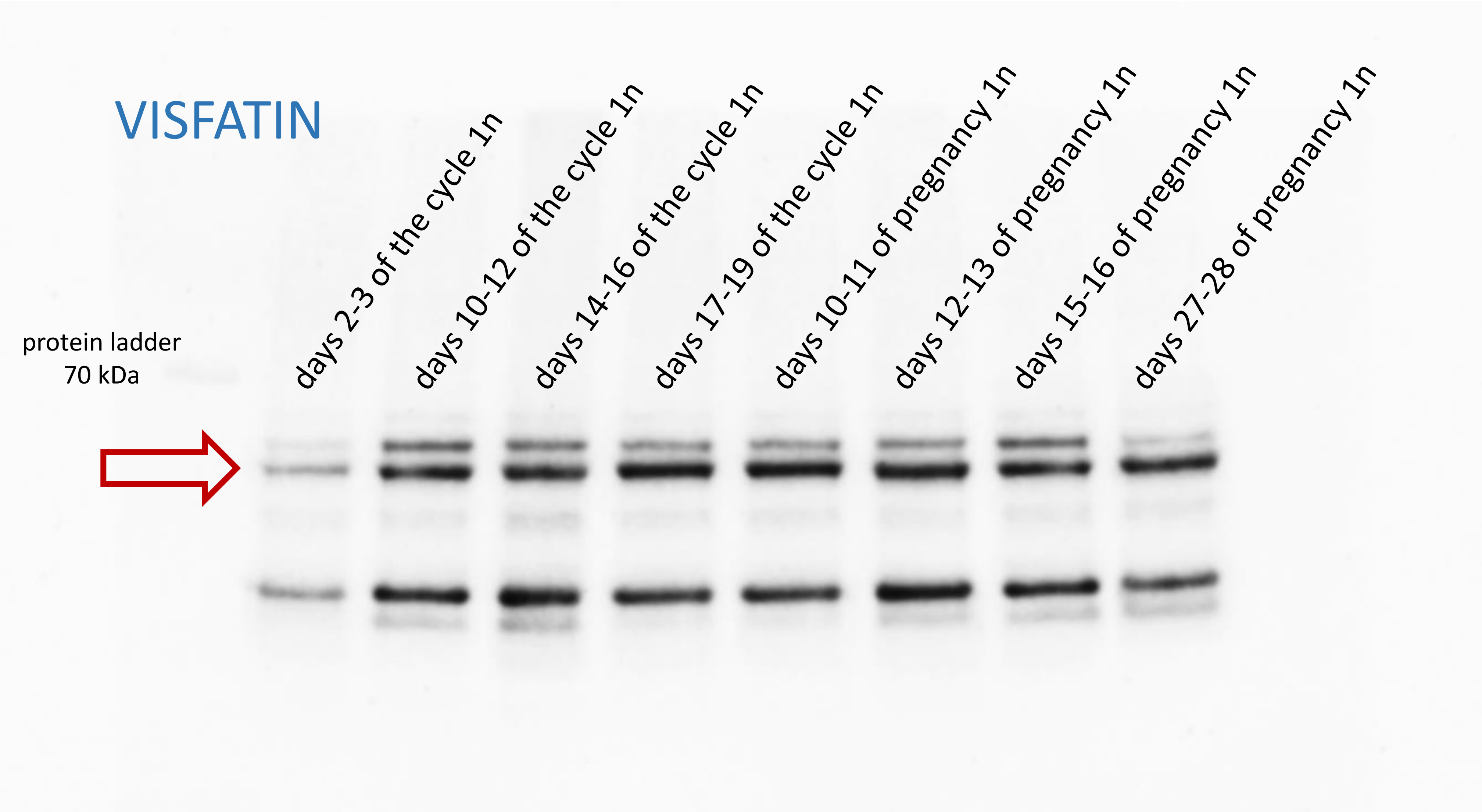

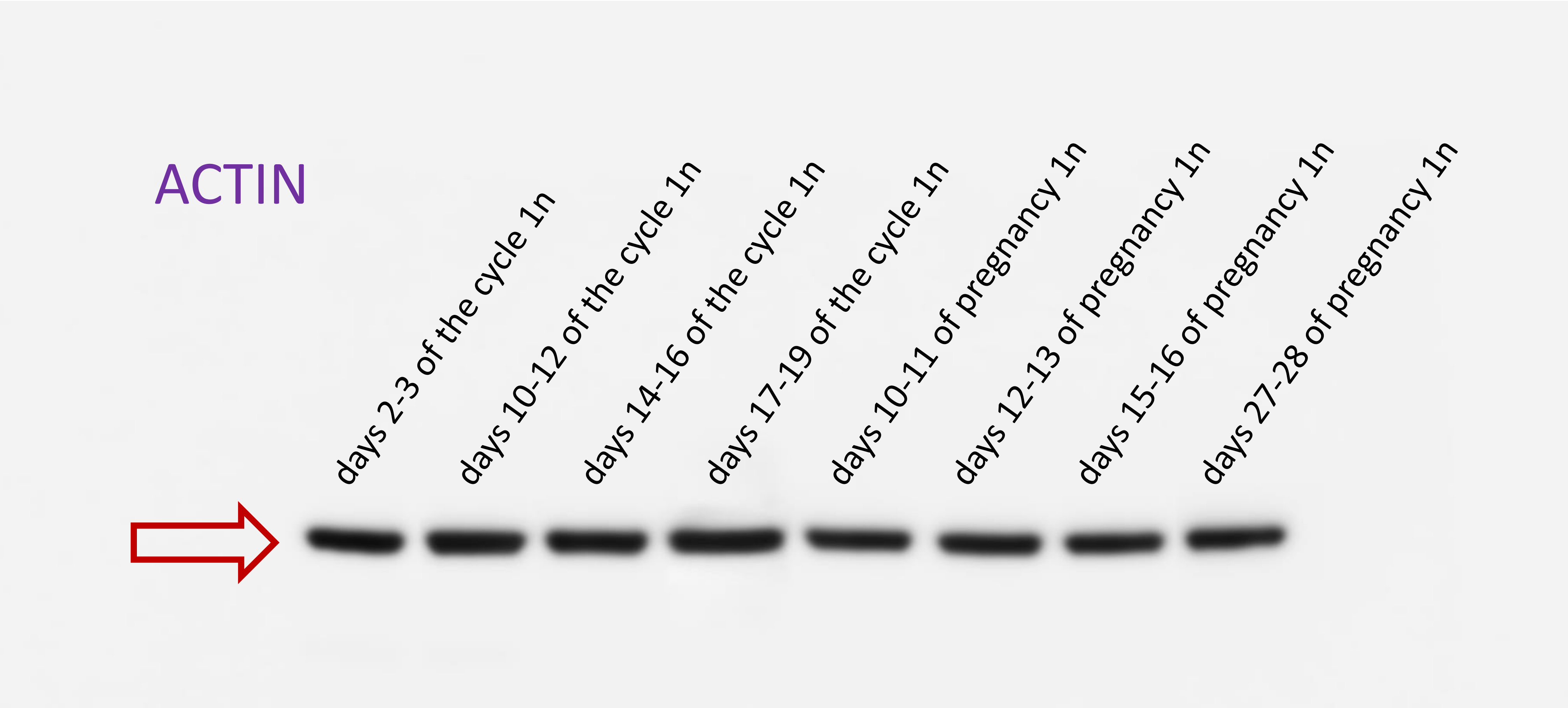

REGULATION OF VISFATIN PROTEIN CONCENTRATION IN THE ANTERIOR PITUITARY ON DAYS 2-3 OF THE CYCLE

VISFATIN

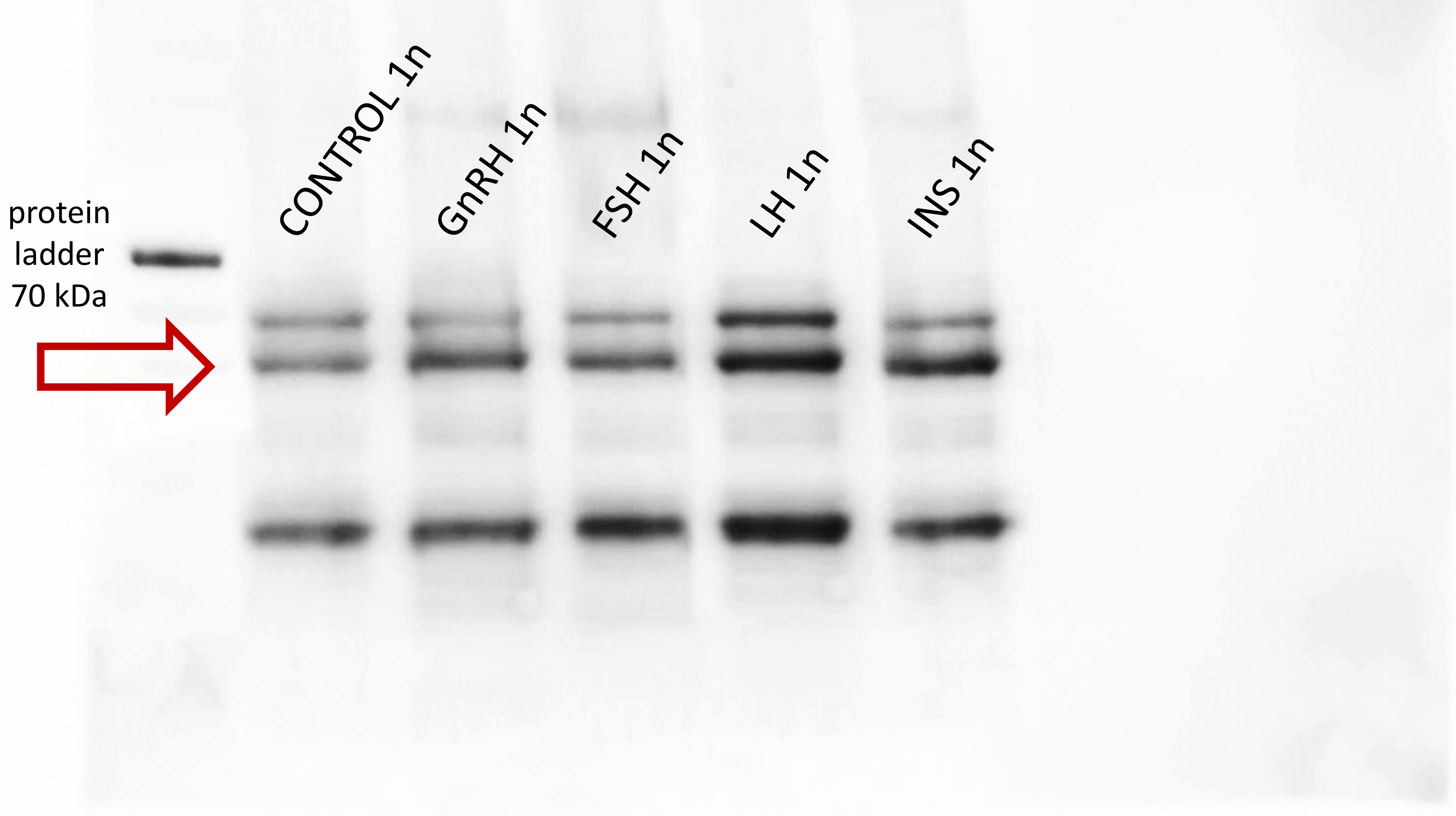

ACTIN

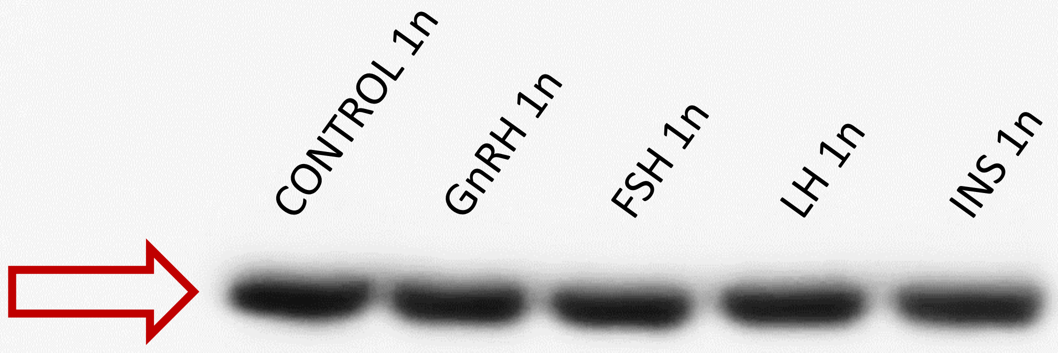

REGULATION OF VISFATIN PROTEIN CONCENTRATION IN THE ANTERIOR PITUITARY ON DAYS 10-12 OF THE CYCLE

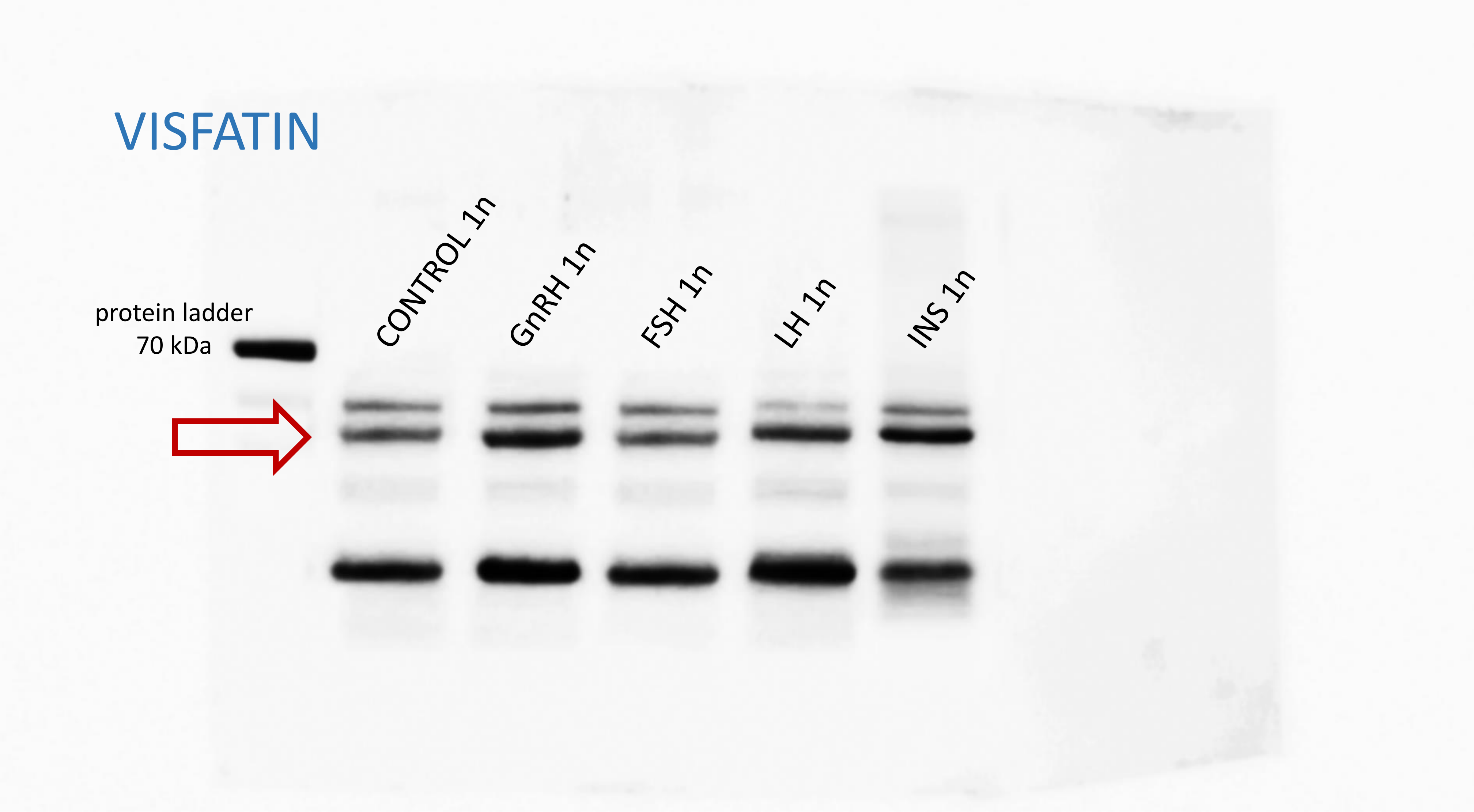

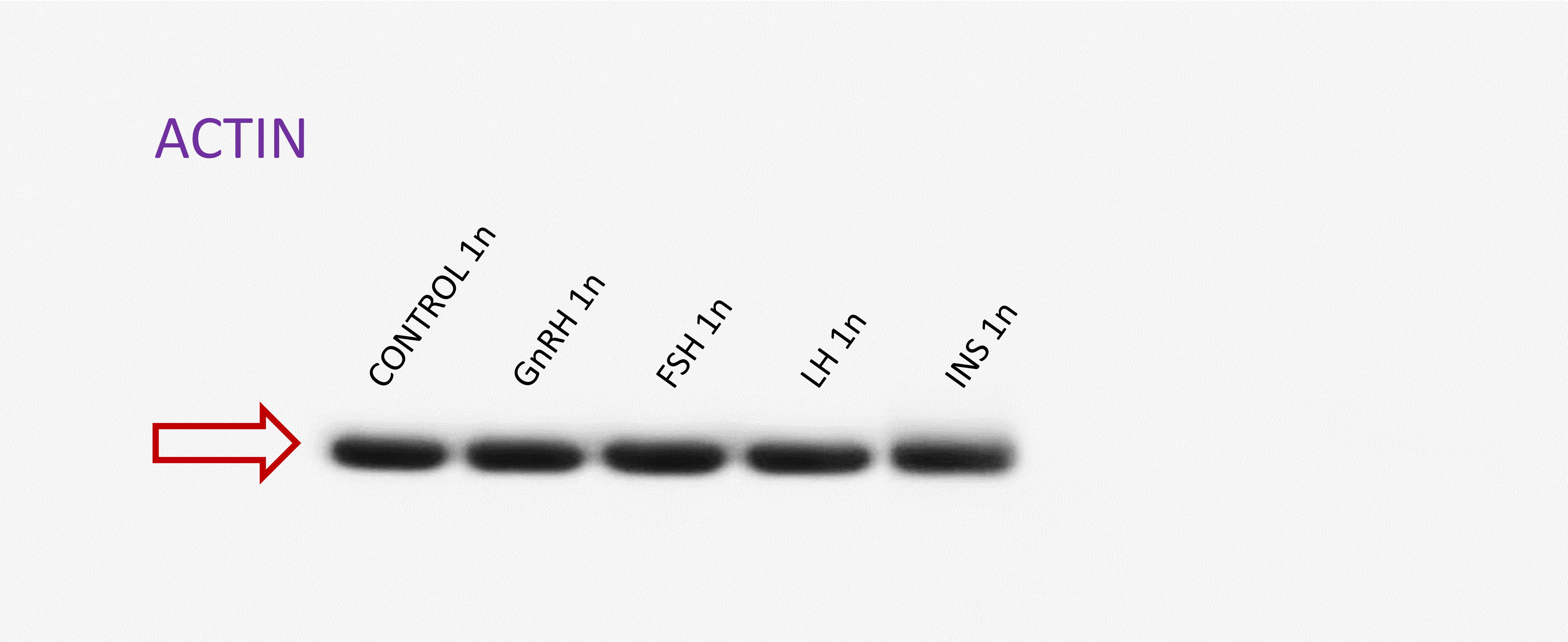

REGULATION OF VISFATIN PROTEIN CONCENTRATION IN THE ANTERIOR PITUITARY ON DAYS 14-12 OF THE CYCLE

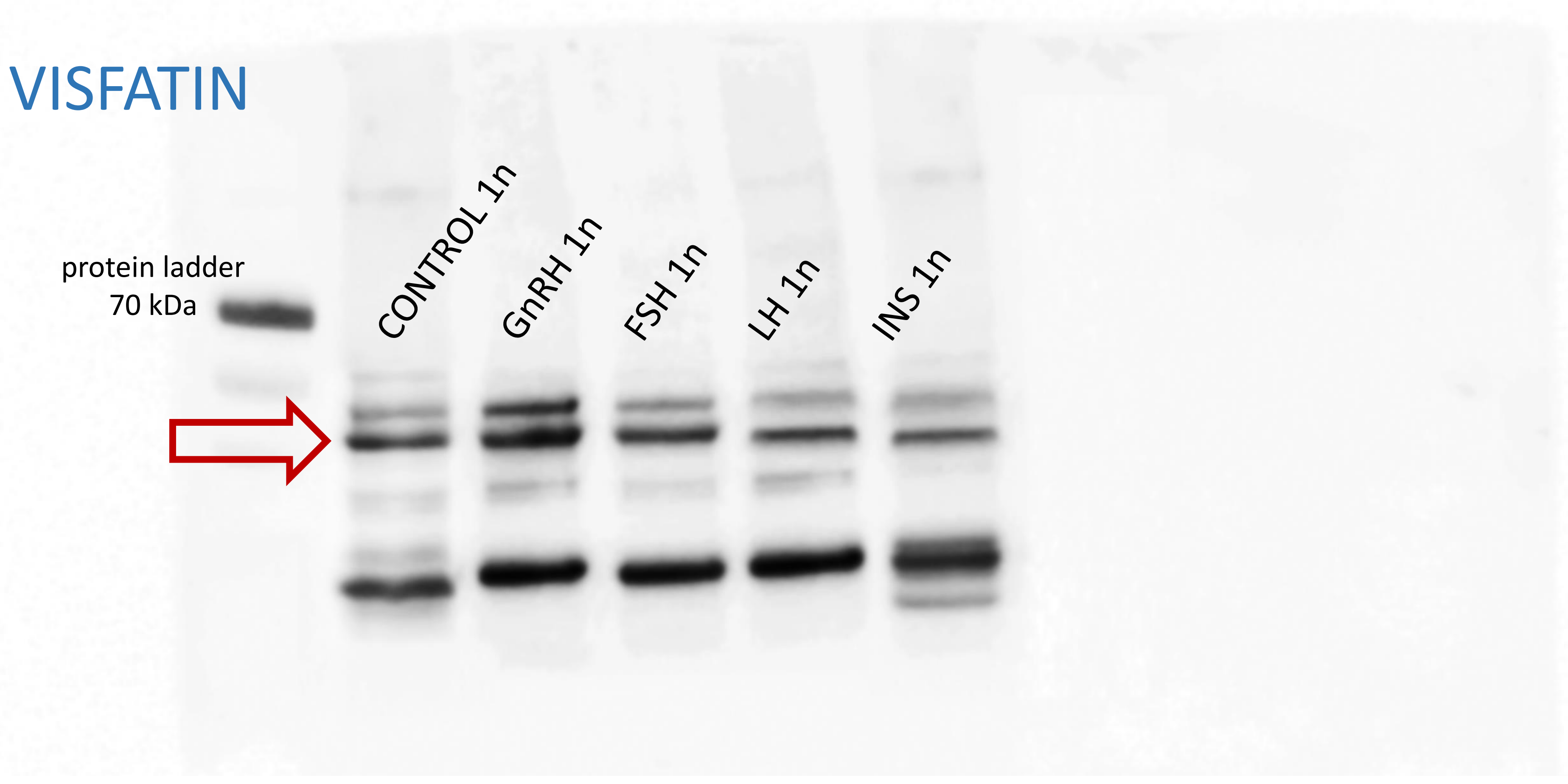

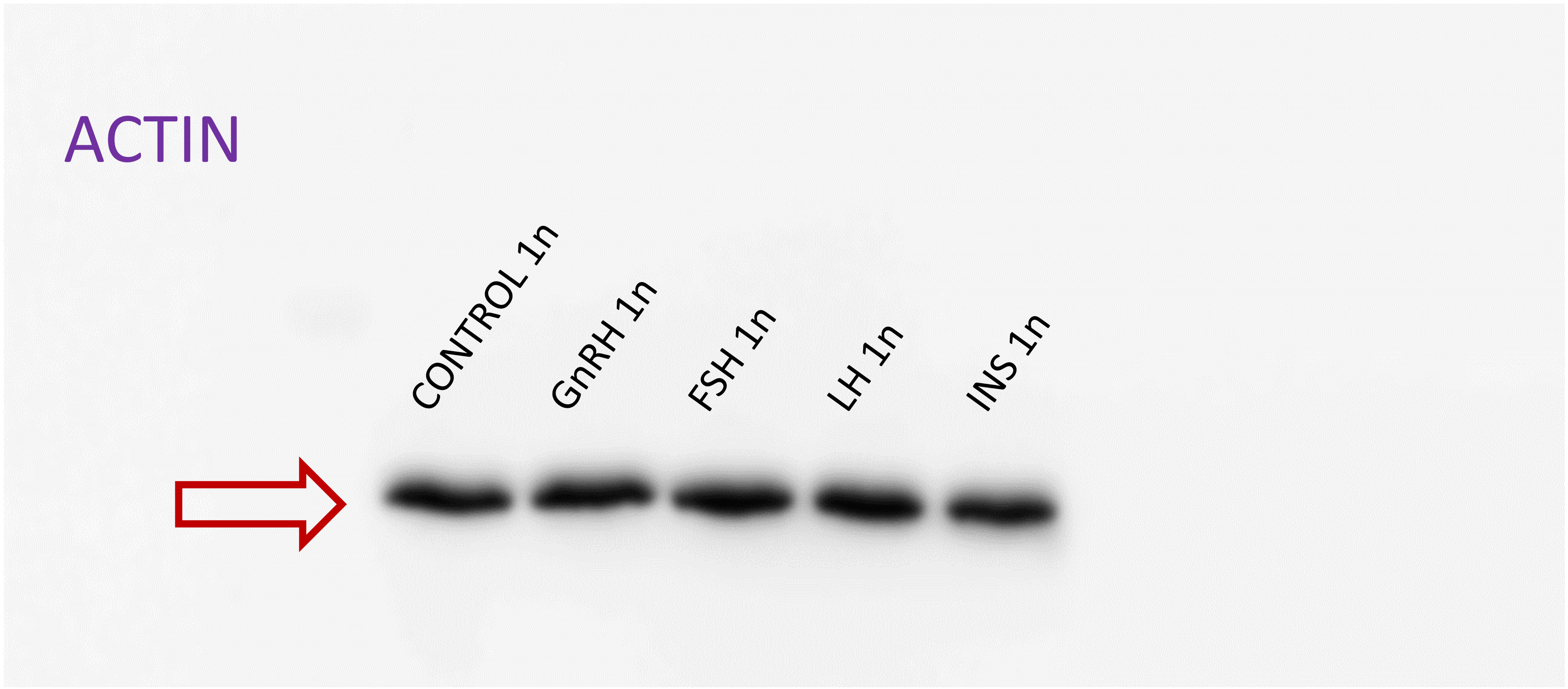

REGULATION OF VISFATIN PROTEIN CONCENTRATION IN THE ANTERIOR PITUITARY ON DAYS 17-19 OF THE CYCLE

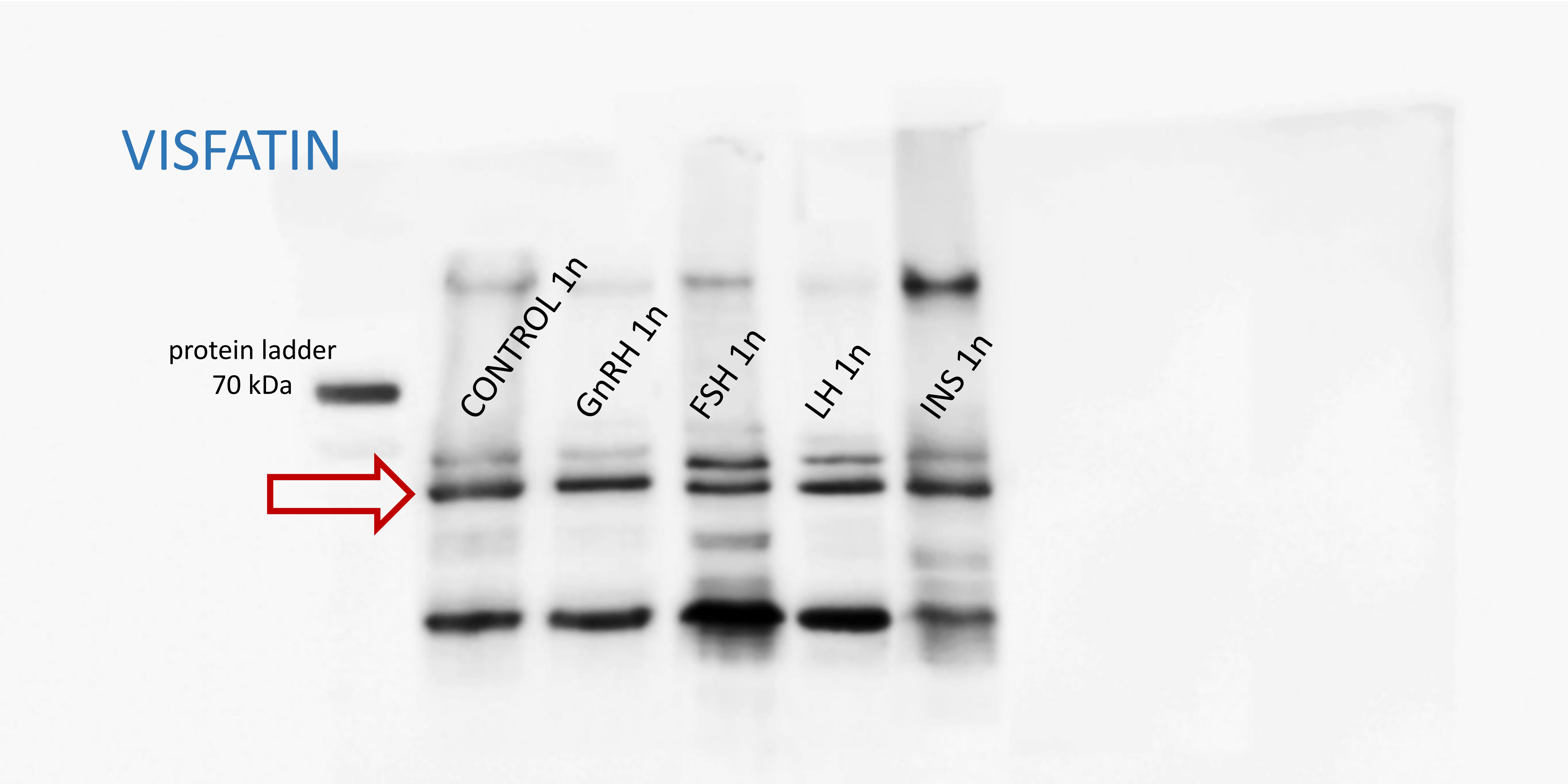

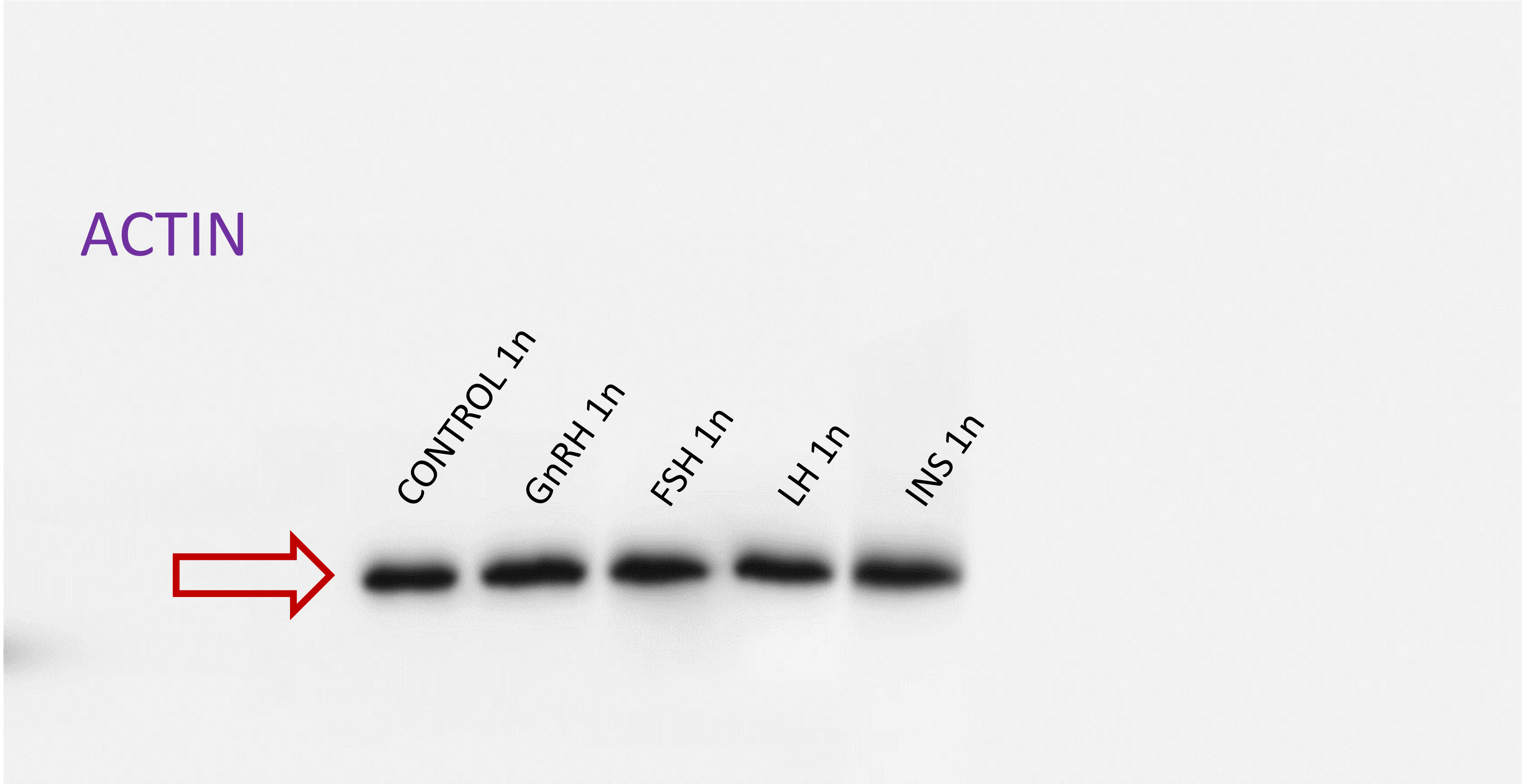

Supplement: Supplementary file 1 — Supplementary Information. [file 41598_2023_45255_MOESM1_ESM.pdf]
